# Hematopoietic Stem Cells in Type 1 Diabetes

**DOI:** 10.3389/fimmu.2021.694118

**Published:** 2021-07-09

**Authors:** Ida Pastore, Emma Assi, Moufida Ben Nasr, Andrea Mario Bolla, Anna Maestroni, Vera Usuelli, Cristian Loretelli, Andy Joe Seelam, Ahmed Abdelsalam, Gian Vincenzo Zuccotti, Francesca D’Addio, Paolo Fiorina

**Affiliations:** ^1^ Division of Endocrinology, ASST Fatebenefratelli-Sacco, Milan, Italy; ^2^ International Center for T1D, Pediatric Clinical Research Center Romeo ed Enrica Invernizzi, DIBIC, Università di Milano, Milan, Italy; ^3^ Nephrology Division, Boston Children’s Hospital and Transplantation Research Center, Brigham and Women’s Hospital, Harvard Medical School, Boston, MA, United States; ^4^ Department of Pediatrics, Buzzi Children’s Hospital, Milan, Italy

**Keywords:** type 1 diabetes, hematopoietic stem cells, autoimmune response, NOD mouse model, genetic modulation

## Abstract

Despite the increasing knowledge of pathophysiological mechanisms underlying the onset of type 1 diabetes (T1D), the quest for therapeutic options capable of delaying/reverting the diseases is still ongoing. Among all strategies currently tested in T1D, the use of hematopoietic stem cell (HSC)-based approaches and of teplizumab, showed the most encouraging results. Few clinical trials have already demonstrated the beneficial effects of HSCs in T1D, while the durability of the effect is yet to be established. Investigators are also trying to understand whether the use of selected and better-characterized HSCs subsets may provide more benefits with less risks. Interestingly, *ex vivo* manipulated HSCs showed promising results in murine models and the recent introduction of the humanized mouse models accelerated the translational potentials of such studies and their final road to clinic. Indeed, immunomodulatory as well as trafficking abilities can be enhanced in genetically modulated HSCs and genetically engineered HSCs may be viewed as a novel “biologic” therapy, to be further tested and explored in T1D and in other autoimmune/immune-related disorders.

## Introduction

Hematopoietic stem cells (HSCs) have been extensively used as an effective therapeutic approach in hematological malignancies and have demonstrated to be safe in human subjects (1). Over the last 10 years, several studies documented the extraordinary immunoregulatory properties of HSCs, which render them a potential useful tool in the fight for immune-mediated diseases (2). Despite being in limited number in the circulating blood of healthy individuals, HSCs are extremely potent and able to suppress the immune system response, as several *in vitro* and *in vivo* studies have shown (2). Based on these premises, the use of HSCs has been tested in numerous autoimmune diseases such as type 1 diabetes (T1D), multiple sclerosis (MS), systemic sclerosis, systemic lupus erythematosus and Chron’s disease, with relevant benefits (3–6). Indeed, HSCs may reset the immune response, thus reshaping the chronic derangement of the immune system to a more self-tolerant state (7, 8). Interestingly, it has been also demonstrated that the bone marrow-derived and blood HSCs are altered in some autoimmune conditions such as T1D and MS, with HSCs being scanty in the circulation and often unable to exploit their immunoregulatory function (9–11). Here we are presenting major advances in the preclinical and clinical studies of HSCs in T1D. We report recent insights coming from novel T1D *in vivo* research and provide an update on the most relevant clinical studies that have been performed by using HSCs in human subjects with T1D. In this perspective, we envision to consider HCSs as a novel “biologic”, which can be personalized and modeled, as a novel relevant therapeutic option in T1D.

## HSCs in Type 1 Diabetes: The Murine Scenario

The rationale behind the use of HSCs in autoimmune disease such as T1D has been extensively studied in the last decade by taking advantage of the NOD mouse model. This mouse spontaneously develops autoimmune diabetes at the age of 12–15 weeks, with severe hyperglycemia (12, 13). However, signs of activation of the immune system against pancreatic islets are already visible at 8–10 weeks of age when the NOD mouse shows insulitis with an abundant T cell infiltrate (12). Over the last two decades, two major HSCs-based strategies have been pursued to prevent the onset of experimental autoimmune diabetes in murine models: (i) HSCs have been infused to induce mixed chimerism and to re-establish the peripheral deletion of autoreactive T cells, (ii) HSCs have been genetically engineered to reshape the immune reservoir and facilitate tolerance towards auto-antigens. The use of HSCs infusion was extremely successful in preventing diabetes onset in NOD mice through the induction of a mixed chimerism. Indeed, a deletion of autoreactive T cells generated at the thymus level (14) as well as the re-establishment of immune tolerance in the periphery were obtained. Furthermore, in the presence of a tolerogenic network between donor Regulatory T cells (Tregs) and host-donor dendritic cells (DCs), costimulatory pathways, particularly PDL-1, play a major role (15). However, the HSC-mediated chimerism, despite effective in reshaping the autoimmune response, requires the use of myeloablative agents/approaches, which may further limit translational applications (16, 17). Given that common polymorphisms exist in MHC class II in T1D patients and in NOD mice, which confer a higher risk of developing T1D, genetically engineering of single HSCs to express the proper and protective MHC class II, held great promises in the new therapies in T1D (18). Indeed, the introduction of new protective MHC class II through lentiviral delivery in HSCs of NOD mice was able to prevent the onset of T1D, mainly through the deletion of autoreactive T cells which did not engage in the MHC class II-mediated response (19, 20). While this approach was again limited by the need of immune ablation for the HSCs infusion, which is feasible in NOD mice but at high risk in humans, it paved the way for exploring genetic engineering of HSCs to better exploit their multiple properties in autoimmunity. *Ex vivo* genetic manipulation of NOD HSCs, to encode proinsulin and transgenically target MHC class II, successfully prevented T1D onset (21, 22). Also, HSCs can be engineered for tolerogenic purposes such as those aimed at inducing tolerance to autoantigens or at replacing genetic alleles associated with increased disease susceptibility (23). In view of this, some studies explored whether HSCs in diabetic NOD mice are altered and might be fixed through genetic engineering or pharmacological modulation. Elevated levels of CXCL12 (SDF-1) in bone marrow-HSCs of NOD mice have been suggested to alter trafficking of HSCs and Tregs in the periphery, thus favoring the onset of T1D (24). The use of ADAM3100, which antagonizes the CXCL12 receptor SDF-1, was associated with increased mobilization of HSCs and T cells, and delayed onset of experimental autoimmune diabetes in NOD mice (24). Recently, a defect in PDL-1 expression has been demonstrated in HSCs of NOD mice, which was associated with a reduced immunomodulatory function (9, 25). Genetic and pharmacological modulation of PDL-1 on HSCs restored the HSCs immunomodulatory properties, reset the immune balance and prevented the onset of T1D. In summary, all the aforementioned studies support the use of *ex vivo* manipulation of HSCs in the NOD mouse model as a successful tool to delay the onset of autoimmune diabetes. Genetic engineering of HSCs has been recently employed in a humanized mouse model in which *ex vivo* manipulated human HSCs successfully restored the development of functional Tregs and rescued the autoimmune IPEX syndrome (26). Recently, the introduction of the NOD-Rag1null IL2rγnull Ins2Akita (NRG-Akita) mouse, a humanized mouse model available in diabetes research which develops spontaneous hyperglycemia, fostered studies in the field (27, 28). This model, in which human immune cells can be infused without being rejected, may be extremely useful in testing the potency of newly genetically engineered human HSCs in the diabetes prevention.

## HSCs in Type 1 Diabetes: The Human Landscape

**Figure 1:**
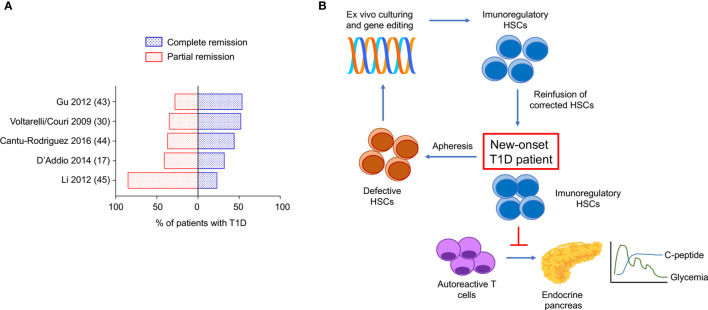
Complete/partial remission of type 1 diabetes obtained with AHSCT in the long-term. Proposed genetic engineered HSC-based approach to target type 1 diabetes. **(A)** Proportion of patients with T1D undergoing AHSCT who achieved complete remission (insulin independence) and partial remission (low dose exogenous insulin requirement) at the latest timepoint analyzed within each clinical study registered in ClinicalTrials.gov available as publication. **(B)** Use of genetically engineered HSCs to target T1D: proposed approach. T1D, type 1 diabetes; AHSCT, autologous hematopoietic stem cell transplantation.

In the last 20 years, autologous hematopoietic stem cells transplantation (AHSCT) has been used in several clinical trials to treat refractory autoimmune disease such as multiple sclerosis (MS), systemic sclerosis (SSc), systemic lupus erythematosus (SLE), Crohn’s disease (CD), type 1 diabetes (T1D) and a range of other immune-mediated disorders (29). With regard to patients with T1D, the use of AHSCT obtained significant insulin independence and a well-preserved glycometabolic control in the short and mid-term follow-up (**Figure 1A** and **Table 1**) (17, 31, 34, 35). Also, an increase in C-peptide levels and C-peptide area under the curve (AUC) measurement were detectable in AHSCT-treated T1D patients as compared to baseline, and only minor adverse events were registered in the mid-term (25, 36). A reduction of the T-helper-1 and T-helper-17 subsets was also observed in the short-term (37). Interestingly, a cost-effectiveness analysis conducted in patients with T1D undergoing AHSCT as compared to patients with T1D remaining on insulin therapy demonstrated that AHSCT provides some benefits over time depending on the duration of preserved glycated hemoglobin levels achieved with AHSCT, but overall being cost-effective for treatment of T1D if the AHSCT lasts from 3 to 8 years (38). Long-term follow-up analyses for AHSCT-treated T1D patients were only reported for a few studies, due to the high number of patients lost at follow-up and the worldwide spreading of the studies, which also accounted for a small sample size, missed randomization process, lack in standardized procedures and enrolment of a heterogenous patients’ population (39). Despite all these limitations, a minor percentage of relapse in the autoimmune disease was evident between 4 and 6 years of follow-up after the AHSCT, which varied among centers and lead few patients to resume insulin treatment (40, 41). Moreover, a subgroup of AHSCT-treated patients exhibited a prolonged remission and remained insulin independent for more than 4 years, thus leading to hypothesize that the response to the AHSCT treatment may differ in patients with T1D (17, 35, 42). Indeed, Malmegrim and Colleagues demonstrated that a different immune profile exists between patients experiencing short and prolonged remission, with the latter exhibiting lower frequencies of effector-memory CD4 T cells and islet-specific autoreactive CD8 T cells, paralleled by a detectable expansion of immunoregulatory T cells (35). Moreover, the favorable outcome of AHSCT in T1D was also associated with a less islet-specific autoreactive immune profile at baseline, thus delineating a subgroup of patients with T1D who may benefit the most from an AHSCT-based strategy (43, 44). This also emphasized the importance of the conditioning regimen, which may need to be employed in association with AHSCT in patients with a high level of autoimmune response. In summary, results of the use of HSC-based approach, primarily the AHSCT, in patients with T1D (**Table 1**), suggest two major observations: (i) AHSCT treatment has to be limited to a subgroup of T1D patients and it requires high-level immunosuppression to obtain long-term effect, and (ii) the immune profile of T1D patients plays a central role in the achievement of long-term insulin-independence when using HSC-based strategies. Therefore, the infusion of a subset of HSCs, rather than the whole HSCs pool, such as in AHSCT, endowed with immunoregulatory properties may provide additional benefits in terms of balancing autoimmunity and achieving the proper clinical and metabolic outcomes.

**Table 1:** Summary of main characteristics, clinical outcomes and results obtained in the clinical studies conducted in T1D and registered in ClinicalTrials.gov.

Clinical Study	N of pts Follow-up Type of study	Clinical outcomes	Main results
Autologous Hematopoietic Stem Cell Transplantation for Early Onset Type 1 Diabetes (NCT00807651)	28 pts T1D 3 years Monocentric prospective	EIR, HbA1c, C-peptide and anti-GAD level	Insulin independence: 53.6% Increased C-peptide level (30)
Safety and Efficacy Study of Autologous Stem Cell Transplantation for Early Onset Type I Diabetes Mellitus (NCT00315133)	23 pts T1D 5 years Monocentric prospective	C-peptide level Morbidity/mortality EIR changes HbA1c level	Insulin independent: 52% Low EIR: 35%; C-peptide AUC increase, HbA1c <7% (31)
Hematopoietic Stem Cell Transplantation in Type 1 Diabetes Mellitus (NCT01121029)	15 pts T1D 3 years Monocentric prospective	EIR C-peptide and HbA1C	Insulin independent: 44% HbA1c decrease: 2.3% Mortality: 0% (32)
Efficacy and Safety Study of Autologous Hematopoietic Stem Cell Transplantation to Treat New Onset Type 1 Diabetes (NCT01341899)	13 pts T1D 4 years Monocentric prospective	C-peptide and HbA1C Islet autoantibodies Immune profile Survival	3/13 pts: no insulin 11/13 pts low EIR, reduced HbA1C low autoantibodies increased C-peptide (33)
Stem Cell Mobilization (Plerixafor) and Immunologic Reset in Type 1 Diabetes (T1DM) (NCT03182426)	60 pts T1D 2 years Monocentric prospective	C-peptide AUC, EIR HbA1C <7% Hypoglycemia Autoantibodies titer	Not available

pts, patients; T1D, type 1 diabetes; AUC, area under the curve.

## Conclusions and Future Directions

The use of HSCs has hold great promises in the treatment of autoimmune diabetes, however, in the last decade. The results obtained in clinical trials with the use of AHSCT in T1D suggest a potential novel approach to treat autoimmune diseases, despite all the aforementioned limitations. The use of a selected subset of HSCs endowed with immunoregulatory properties, without the need of additional immunosuppressive agents remains unexplored so far and deserves more investigation and testing from the scientific community. Patients with T1D who may benefit the most from this therapeutic approach need to be carefully identified, probably based on disease stages, degree of cellular and humoral autoimmune response, presence or not of diabetic ketoacidosis (17). The recent findings on the use of teplizumab in patients at risk for T1D (45) confirmed that immune ablation aimed at preventing T1D onset is a hot topic. HSCs, endowed with immunomodulatory properties, may offer a potent immunoregulatory effect without inducing T lymphocytes depletion, which is commonly observed with teplizumab. Indeed, several studies demonstrated that in absence of “healthy” HSCs central tolerance may be difficulty obtained. Autoimmune disorders, particularly type 1 diabetes, are associated with altered HSCs, which fail in exerting their immunomodulatory properties. Strategies aimed at targeting this defect successfully delayed diabetes onset in murine models. Feasibility and effectiveness in of the *ex vivo* manipulation and genetic engineering of HSCs are well-established in mouse models, while studies on safety for translational purposes are still required. In view of this, the use of humanized mouse model may accelerate the translation from murine experiments to human studies. The outstanding results collected in the past and ongoing clinical trials are encouraging in pursuing the research around the use of genetic engineered-HSCS in type 1 diabetes. Therefore, in our opinion, genetic modulation to reset HSCs physiological function, may find an interesting field of application not only in type 1 diabetes (**Figure 1B**) but in other autoimmune conditions too. Finally, in the era of the development of biologic therapy to treat immune-mediated diseases, we envision genetically engineered HSCs as a novel “biologic” agent and a “natural immunosuppressant” to be considered in the portfolio of alternative therapeutic options in type 1 diabetes and autoimmune diseases.

## Author Contributions

IP and EA wrote the paper. AB, MB, AM, CL, VU, AS, and AA, collected clinical and preclinical data. GZ edited the paper. FD’A and PF conceived the idea, wrote and edited the paper. All authors contributed to the article and approved the submitted version.

## Funding

FD is supported by SID Lombardia Grant and by EFSD/JDRF/Lilly Programme on Type 1 Diabetes Research 2019. PF is supported by the Italian Ministry of Health grant RF-2016-02362512 and by the Linea-2 2019 funding from Università di Milano. We thank the “Fondazione Romeo e Enrica Invernizzi” for extraordinary support.

## Conflict of Interest

PF and MB hold a patent of modulated HSCs and founded Altheia Science.

The remaining authors declare that the research was conducted in the absence of any commercial or financial relationships that could be construed as a potential conflict of interest.
